# Ascertaining the burden of invasive *Salmonella* disease in hospitalised febrile children aged under four years in Blantyre, Malawi

**DOI:** 10.1371/journal.pntd.0007539

**Published:** 2019-07-17

**Authors:** Chisomo L. Msefula, Franziska Olgemoeller, Ndaru Jambo, Dalitso Segula, Trinh Van Tan, Tonney S. Nyirenda, Wilfred Nedi, Neil Kennedy, Matthew Graham, Marc Y. R. Henrion, Stephen Baker, Nicholas Feasey, Melita Gordon, Robert S. Heyderman

**Affiliations:** 1 Pathology Department, College of Medicine, University of Malawi, Blantyre, Malawi; 2 Malawi-Liverpool-Wellcome Trust Clinical Research Programme, College of Medicine, University of Malawi, Blantyre, Malawi; 3 Department of Paediatrics, Queen Elizabeth Central Hospital, College of Medicine, University of Malawi, Blantyre, Malawi; 4 University of Liverpool, Liverpool, United Kingdom; 5 Department of Internal Medicine, Queen Elizabeth Central Hospital, College of Medicine, University of Malawi, Blantyre, Malawi; 6 The Hospital for Tropical Diseases, Wellcome Trust Major Overseas Programme, Oxford University Clinical Research Unit, Ho Chi Minh City, Vietnam; 7 Centre for Medical Education, Queens University, Belfast, United Kingdom; 8 Liverpool School of Tropical Medicine, Liverpool, United Kingdom; 9 The Department of Medicine, The University of Cambridge, Cambridge, United Kingdom; 10 Division of Infection & Immunity, University College London, London, England, United Kingdom; University of Colorado Health Sciences Center, UNITED STATES

## Abstract

Typhoid fever is endemic across sub-Saharan Africa. However, estimates of the burden of typhoid are undermined by insufficient blood volumes and lack of sensitivity of blood culture. Here, we aimed to address this limitation by exploiting pre-enrichment culture followed by PCR, alongside routine blood culture to improve typhoid case detection. We carried out a prospective diagnostic cohort study and enrolled children (aged 0–4 years) with non-specific febrile disease admitted to a tertiary hospital in Blantyre, Malawi from August 2014 to July 2016. Blood was collected for culture (BC) and real-time PCR after a pre-enrichment culture in tryptone soy broth and ox-bile. DNA was subjected to PCR for *invA* (Pan-*Salmonella*), *staG* (*S*. Typhi), and *fliC* (*S*. Typhimurium) genes. A positive PCR was defined as *invA* plus either *staG* or *fliC* (CT<29). IgM and IgG ELISA against four *S*. Typhi antigens was also performed. In total, 643 children (median age 1.3 years) with nonspecific febrile disease were enrolled; 31 (4.8%) were BC positive for *Salmonella* (n = 13 *S*. Typhi, n = 16 *S*. Typhimurium, and n = 2 *S*. Enteritidis). Pre-enrichment culture of blood followed by PCR identified a further 8 *S*. Typhi and 15 *S*. Typhimurium positive children. IgM and IgG titres to the S. Typhi antigen STY1498 (haemolysin) were significantly higher in children that were PCR positive but blood culture negative compared to febrile children with all other non-typhoid illnesses. The addition of pre-enrichment culture and PCR increased the case ascertainment of invasive *Salmonella* disease in children by 62–94%. These data support recent burden estimates that highlight the insensitivity of blood cultures and support the targeting of pre-school children for typhoid vaccine prevention in Africa. Blood culture with real-time PCR following pre-enrichment should be used to further refine estimates of vaccine effectiveness in typhoid vaccine trials.

## Author summary

There are increasing reports of typhoid fever epidemics in sub-Saharan Africa frequently affecting young adults and children aged between 5 and 16 years. In Asia where typhoid is hyperendemic, children aged 0 to 4 years also have a high burden of typhoid fever. Diagnosis of typhoid in young children is particularly a challenge because collection of adequate blood sample for testing is not always possible and the Salmonella bacterial load is low. Established methods of diagnosis such as blood culture and serology have low sensitivity. This study has used a combination of blood culture and pre-enrichment culture followed by PCR to improve ascertainment of the burden of both nontyphoidal Salmonella disease and typhoid fever in Malawian children, aged 0 to 4 years. We found that diagnosis with blood culture and pre-enrichment culture followed by PCR together added 94% more of nontyphoidal Salmonella and 62% more of Salmonella Typhi than blood culture alone. Where blood culture was negative but Salmonella-specific PCR was positive we have validated our results using Haemolysin (STY1498)-based serology. There are ongoing typhoid vaccine efficacy trials in Africa and Asia. The findings from this study will inform future estimates of vaccine effectiveness.

## Introduction

Both *Salmonella* Typhi and nontyphoidal Salmonellae remain prominent contributors to the large burden of bloodstream infection (BSI) in sub-Saharan Africa (sSA) [1–4]. Until recently nontyphoidal serovars *Salmonella* Typhimurium and *Salmonella* Enteritidis were the most prevalent in sSA, mainly affecting young children and HIV-infected adults [5–7]. Outbreaks of typhoid fever are now being reported across sSA [1, 8–11], largely caused by a multidrug resistant *S*. Typhi genotype 4.3.1 [12–14]. In Malawi where surveillance for bloodstream infections has been conducted for over 20 years, *S*. Typhi has become one of the commonest blood culture isolates amongst hospitalized febrile adults and children [1, 15]. In this context, ineffective, commonly available antimicrobials and inadequate diagnostic tools with poor sensitivity and results turn-around time, hamper the identification, management and control of both iNTS and typhoid fever.

Blood culture identifies 40 to 80% of cases of invasive Salmonella disease [16]. Limitations of culture arise due to low numbers of bacteria in blood, requiring large volume samples that are not feasible from young children [17]. Prior antimicrobial use may further compromise the diagnostic yield. Currently available, commercial serological tests detecting antibody against *S*. Typhi antigens have limited sensitivity and poor specificity [18–20]. A number of PCR methods for diagnosis of *Salmonella* have been developed. The inclusion of a culture step prior to nucleic acid amplification has been suggested to increase sensitivity [21, 22], but this has not been evaluated in the field.

Studies from countries in Asia with endemic typhoid suggest a considerable disease burden in young children, including children aged under 4 years [3, 23–25]. If this is also the case in sSA, this could influence the age at which vaccination might be implemented within the National vaccination expanded programme on immunisation [26]. In this study, we adopted real-time PCR with a pre-enrichment culture step alongside standard blood culture to improve case ascertainment of invasive *Salmonella* disease in children aged under 4 years in Malawi.

## Methods

### Ethics statement

The College of Medicine Research Ethics Committee approved the study, approval number P.08/13/1445. Written informed consent was sought from the parents or guardians before enrolment of participants into the study.

### Participant enrolment

Recruitment of participants into the study was nested within the pre-existing sentinel surveillance for bacterial infections at Queen Elizabeth Central Hospital (QECH) and the Malawi-Liverpool-Wellcome Trust Clinical Research Programme, Blantyre, Malawi. Children with non-specific febrile illness (temperature ≥38°C or a history of fever) aged between 0 and 4 years old presenting to QECH were recruited. QECH is the largest government hospital in Malawi, providing free healthcare to the district of Blantyre with a population of approximately 1.3 million and referrals from the Southern region [27]. Recruitment was from August 2014 to July 2016 covering three dry seasons and two rainy seasons. 5 mL blood was drawn from each child; 2 mL for culture, 2 mL for real-time PCR and 1 mL for serology. At six weeks in convalescence further 1 mL of blood was drawn and plasma was extracted for serology. Unless invasive bacterial infection was strongly suspected, children with a positive RDT or malaria blood film were not enrolled into the study.

### Blood culture processing

Blood cultures were processed using the BacT/Alert automated system (Biomerieux, France), at the Malawi-Liverpool-Wellcome Trust Clinical Research Laboratories [1]. Bacteria were isolated and identified using previously described standard microbiological procedures [1]. A minimum of four days, including sub-culturing, biochemical testing and latex agglutination testing, was necessary to generate a definitive report of the diagnosis.

### Pre-enrichment

A modified pre-enrichment procedure for *Salmonella* was adopted [28]. A maximum of 2 mL of blood was added to 8 mL of tryptone soy broth mixed with 3% Ox-gall powder (TSB/Ox-gall) and incubated overnight at 37°C. Blood samples with <2 mL were added to 8 mL TSB/Ox-gall medium, topped up to 10 mL with sterile distilled water.

### DNA extraction and real-time PCR

Pre-enriched blood samples were centrifugated in a macrocentrifuge at 3000 *x g* and the supernatant pipetted out leaving approximately 200 μl to resuspend the pellets. DNA from blood, including bacterial genomic DNA, was extracted following the UltraClean BloodSpin Kit (MO BIO Laboratories CA USA) extraction protocol. DNA was eluted in a final volume of 100 μl and stored at -20°C. A 40-cycle real-time PCR was performed using a mastermix of Platinum Quantitative UDG reagents on an ABI 7500. Three primer pairs (Table 1) designed using Primer Quest, were added in monoplex reactions (S1 Table) to detect the presence of either *S*. Typhi or *S*. Typhimurium. A pan-*Salmonella* primer pair targeting *invA* was designed using sequences from *S*. Typhimurium ST313 D23580 (Accession FN424405) and *S*. Typhi CT18 (Accession AL513382). A primer pair specifically identifying *S*. Typhimurium was designed against the flagellin gene *fliC* and *S*. Typhi was detected using a primer pair targeting the fimbrial gene *staG*.

**Table 1:** Primer and probe sequences.

Primer	Sequence		Source
invA	Forward	AGCGTACTGGAAAGGGAAAG	This study
	Probe	FAM-TTACGGTTCCTTTGACGGTGCGAT-BHQ1	
	Reverse	CACCGAAATACCGCCAATAAAG	
staG	Forward	CCGACCAAGTTCCAGATCAA	This study
	Probe	VIC-TGGCCAGTAATAATGTCGGGACGA-BHQ1	
	Reverse	GTTGGTTAGTAGCGAGGTGTT	
fliC	Forward	TGCTGATTTGACAGAGGCTAAA	This study
	Probe	FAM-TGTTACCGGCACAGCATCTGTTGT-BHQ1	
	Reverse	TCGCCTACCTTAACTGCTAAAC	

### Establishing a cycle threshold cut-off

A positive PCR result was assigned to a sample only if the pan-*Salmonella* (*invA*) amplification was simultaneously positive with either one of the serovar-specific amplifications (*fliC* or *staG*) within a specified cycle threshold (CT) cut-off. The CT-value cut-off was determined using a combination of three approaches. Firstly, the primer pairs were tested against DNA extracted from serially diluted cultures of *S*. Typhi, *S*. Typhimurium, *Staphylococcus aureus*, *E*. *coli*, *Klebsiella pneumoniae*, *Micrococcus* spp., and *Bacillus* spp (S2 Table). Five replicates were prepared for each organism. A cut-off was set to exclude all CT-values observed for non-specific target amplifications. Secondly, the limit of target detection was established for the three primer pairs by running the real-time PCR assay on five replicates of serially diluted cultures of *S*. Typhi and *S*. Typhimurium. Thirdly, a receiver operating characteristic (ROC) curve was constructed to assess the cut-off value that maximises sensitivity and specificity of the PCR amplification, considering the blood culture result as a true positive.

### *S*. Typhi serology

Serology for *S*. Typhi (Table 2), with previously identified serodiagnostic antigens [29, 30] and S. Typhi Vi polysaccharide antigen [31], was conducted to validate the PCR amplification data in blood culture negative samples. Sera from febrile children acutely ill and plasma at six weeks in convalescence were analysed. Archived serum samples from healthy Malawian children aged 0 to 4 years (median age 10 months) were included in the analysis as controls [32]. IgM and IgG titres in acute and convalescent plasma samples and in healthy control sera were measured using a previously described ELISA with 3 purified protein antigens STY1498 (Haemolysin), STY1479 (a possible ATP binding protein), STY1886 (cytolethal distending toxin subunit B homolog) and S. Typhi Vi polysaccharide antigen.

**Table 2:** *Salmonella* Typhi antigens for ELISA confirmation of blood culture negative/ PCR positive samples.

Antigen ID	Annotation
STY1498	Haemolysin
STY1479	Possible ATP binding protein
STY1886	Cytolethal distending toxin subunit B homolog
VI	Vi polysaccharide

### Statistical analysis

Fisher’s exact test and Chi-squared test were used to determine whether clinical characteristics (fever, vomiting and diarrhoea), hospital admission and reported prior use of antibiotics were related to detection of Salmonella by both culture and PCR. Statistical significance was defined as *P* < 0.05, although exact p-values were reported. Pearson’s correlation coefficient was used to analyse the association between blood sample volume and cycle threshold value on PCR. Antibody (IgG and IgM) titre differences between *S*. Typhi positive and negative groups were log-transformed and tested using two-tailed two-sample t-tests and we also looked for differential clustering between these 2 groups using multidimensional scaling and principal component analysis. Analyses were performed using IBM Corp. Released 2011. IBM SPSS Statistics for Windows, Version 20.0. Armonk, NY: IBM Corp, GraphPad prism version 7.0 and R v3.5.1.

## Results

### Study population

**Fig 1:**
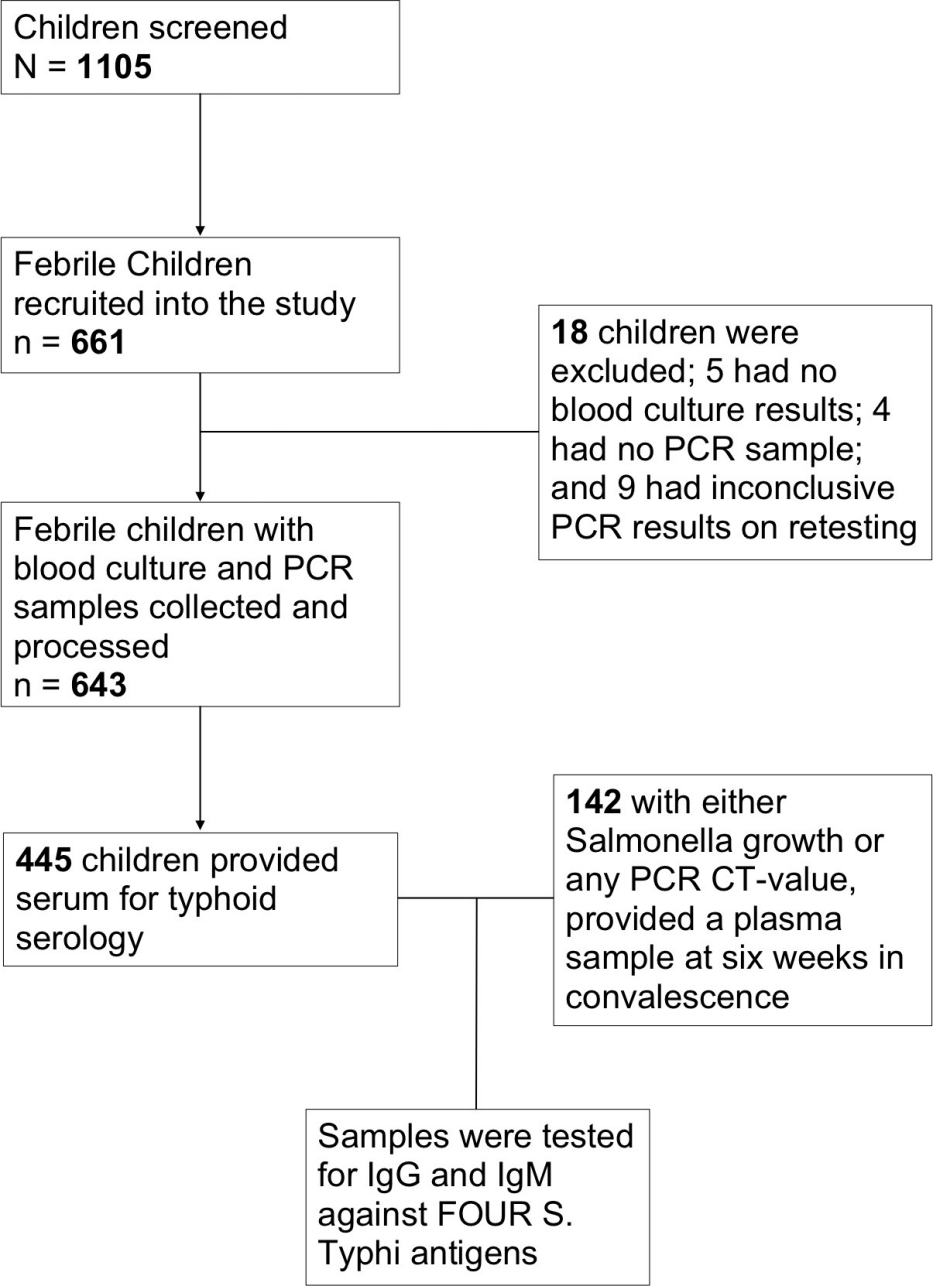
Flowchart of participant recruitment. Numbers of children screened, recruited and processed on PCR, blood culture and serology are provided.

A total of 661 children aged less than 4 years (median age 15.9 months; range 1–48 months; 357 males), with non-specific febrile disease were recruited at QECH over the study period. Eighteen children were excluded from the analysis; 5 had no blood culture results, 4 had no PCR sample, and 9 had inconclusive PCR results on re-testing (Fig 1).

The baseline characteristics of the 643 children in these analyses are presented in Table 3.

**Table 3:** Characteristics of 643 children ≤ 4 years.

Characteristic	Children ≤ 4yrs n (%)
Number	643
Sex, male	351 (54.6)
Age, months, median [IQR]^ ^	15.8 [8.9–28]
1–8.9	165 (25.5)
9–16.9	172 (26.7)
17–24.9	114 (17.7)
25–48	192 (29.7)
Vomiting	261/641 (40.7)
Diarrhoea	227/641 (35.4)
Malaria positive by RDT or Blood Film Microscopy	20/640 (3.1)
Recruitment by season, wet	360 (56.0)
Hospital admission	252/639 (39.4)
Prior reported antibiotic use	288/642 (44.9)

### Real-time PCR and CT-value cut-off

**Fig 2:**
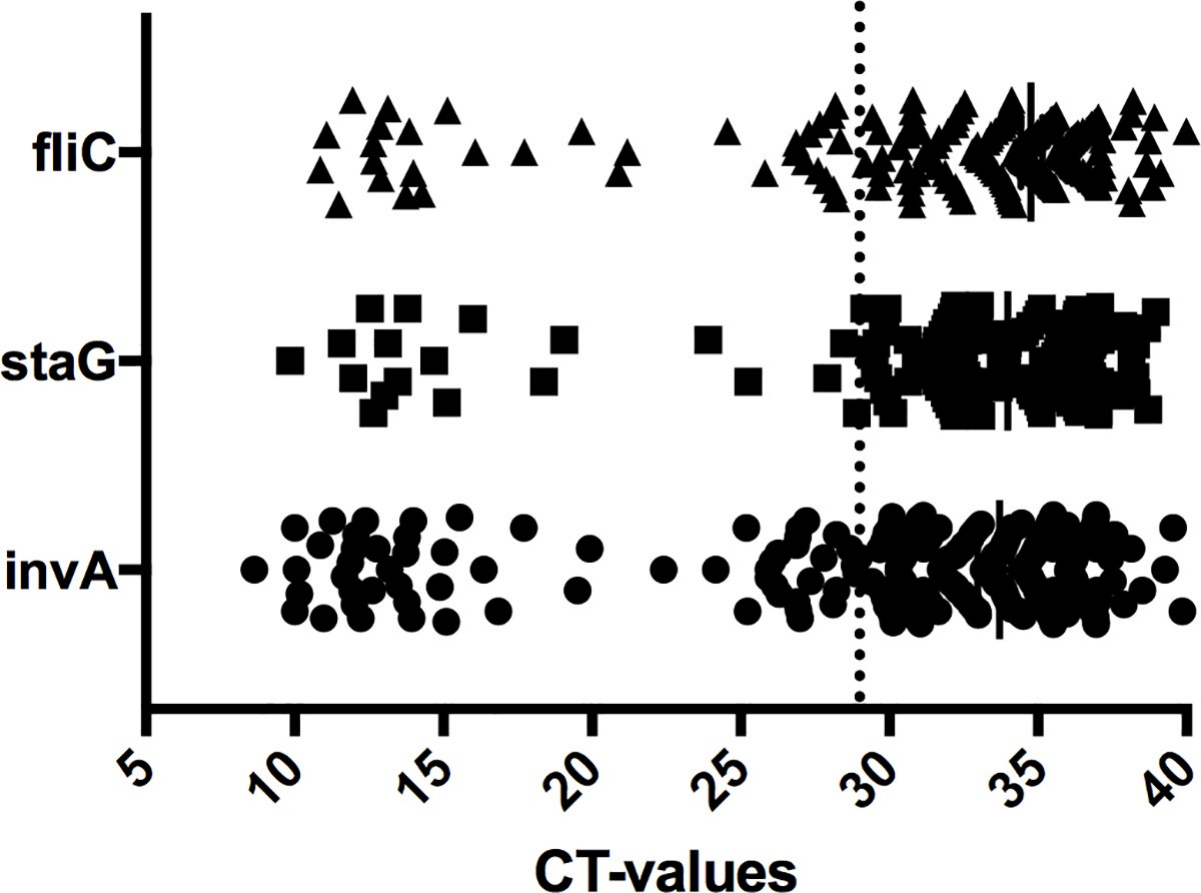
Bimodal distribution of CT-values for primers invA, fliC and staG. Graph shows the distribution of CT-values for the salmonella pan primer and S.Typhi and S.Typhiurium specific primers with a clear distinction between PCR positives and negatives.

**Fig 3:**
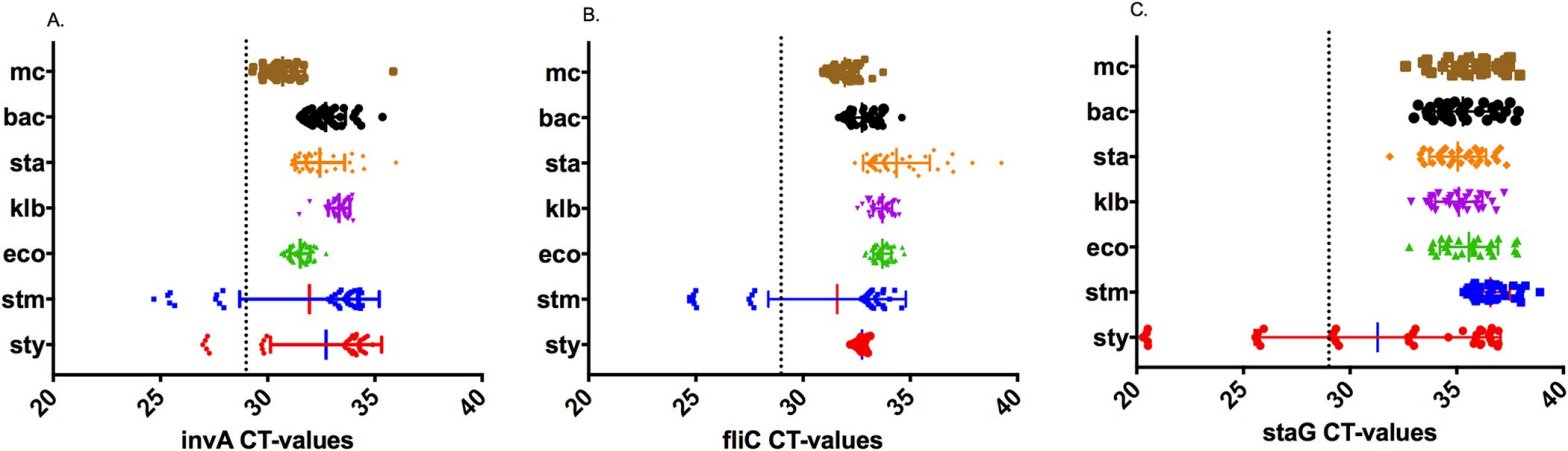
**CT cut-off set to demarcate non-specific amplification signals for primers invA (a), fliC (b) and staG (c)**. Graph shows CT-values from serial dilutions of contaminants; Micrococci (mc), Bacillus (bac), and pathogens; *S*. Typhi (sty), *S*. Typhimurium (stm), *S*. *aureus* (sta), Klebsiella (klb), and *E*. *coli* (eco).

Real-time PCR amplifications were generated in 323 pre-enriched blood samples for the pan-*Salmonella* primers, in 234 for the *S*. Typhimurium specific primers, and for 168 of the *S*. Typhi specific primers, and the remaining 320 samples were negative. The CT values ranged from 8.643 to 39.857 for the pan-*Salmonella* primers (median = 33.713, interquartile range (IQR) 30.476–35.662), from 10.855 to 39.9997 for the *S*. Typhimurium specific primers (median = 34.765, IQR 32.237–36.066), and 9.849 to 38.868 for the *S*. Typhi specific primers (median = 33.978, IQR 31.872–36.708). A plot of the CT-values generated bimodal distributions for each of the three amplifications (Fig 2). A cut-off was set at CT = 29 because non-specific PCR amplifications generated in validation experiments clustered above a CT value of 29 (Fig 3). An ROC curve showed that a CT value of 29 cut-off maximised sensitivity and specificity of the respective PCR primer pairs to identify *S*. Typhi (invA: Sensitivity = 84.6%, Specificity = 92.9%; staG: Sensitivity = 76.9%, Specificity = 98.7%) and *S*. Typhimurium (invA: Sensitivity = 68.8%, Specificity = 92.8%; fliC: Sensitivity = 68.8%, Specificity = 97.0%) (S1 Fig and S3 Table)

### Blood culture and PCR diagnosis

A positive blood culture was recorded in 15.4% (99/643) of the recruited children. Nine pathogens were isolated including the *Salmonella enterica* serovars Typhi, Typhimurium and Enteritidis (Table 4). *S*. Typhimurium was the major NTS serovar with 88.9% (16/18) of NTS isolates. Thirteen of the 643 children had an *S*. Typhi infection; all were older than one year of age and 84.6% (11/13) of them were between 2 and 4 years old. Contaminated blood culture growth was present in 8.9% (57/643) of the study participants. Real-time PCR with pre-enrichment added an additional 8 *S*. Typhi infections and 15 additional *S*. Typhimurium infections. Three *S*. Typhi and 5 *S*. Typhimurium cases had a positive blood culture but were negative by real-time PCR (Table 5). In the study population, the combination of blood culture and real-time PCR with pre-enrichment detected *S*. Typhimurium and *S*. Typhi positivity frequencies of 4.8% (31/643) and 3.3% (21/643) respectively.

**Table 4:** Blood culture growth observed in the study population.

Blood culture growth	n (%)
**Pathogens:**	
*E*. *coli*	3 (0.5)
*Enterobacter* spp	1 (0.2)
*Pantoea* spp	1 (0.2)
*Pseudomonas aeruginosa*	1 (0.2)
*Salmonella* Enteritidis	2 (0.3)
*Salmonella* Typhi	13 (2.0)
*Salmonella* Typhimurium	16 (2.5)
*Staphylococcus aureus*	3 (0.5)
*Streptococcus pyogenes*	2 (0.3)
**Contaminants:**	
α-haemolytic Streptococci	4 (0.6)
*Bacillus* spp.	10 (1.5)
Coagulase negative Staphylococcus	22 (3.4)
Diphtheroids	4 (0.6)
*Micrococcus* spp	17 (2.6)
**No growth**	544 (84.6)
**Total**	**643 (100)**

**Table 5:** PCR performance in relation to Blood culture.

		Blood culture		Sensitivity (%)	Specificity (%)	PPV (%)	NPV (%)
		**+**	**-**				
S.Typhi PCR	**+**	10	8	76.9	98.7	55.6	99.5
	**-**	3	622				
S. Typhimurium PCR	**+**	11	15	68.8	97.6	42.3	99.2
	**-**	5	612				

### Clinical characteristics, antimicrobial use and *Salmonella* detection

Non-specific clinical presentations including fever, vomiting and diarrhoea were common and there was no association between clinical signs or symptoms and detection of either *S*. Typhi or *S*. Typhimurium infection (*P* range 0.27 to 0.79). However, hospital admission was more frequent in children with *S*. Typhimurium infection (23/31; 74.1%) than those with typhoid fever (8/21; 38.0%) (*P* = 0.02). Reported prior-antibiotic use (288/642; 44.9%) was not significantly associated with lack of growth of either *S*. Typhi (*P* = 0.22) or nontyphoid Salmonella (*P =* 0.93).

### Real-time PCR and sample volume

Blood sample volumes were recorded in 619 of the 643 recruited children. The targeted volume of blood (2 mL) for real-time PCR was achieved in 100 /619 (16.2%) children. The minimum volume of blood sample collected for PCR after venesection for blood culture was 100μl (median = 1,200μl, IQR 1,000μl– 1,725μl). There was no correlation between the CT value and the volume of the pre-enriched blood sample for the pan-primer (*P* = 0.35, *r* = .05), *S*. Typhi specific primer (*P* = 0.59, *r* = .04), and *S*. Typhimurium specific primer (*P* = 0.13, *r* = .10) (S2 Fig).

### Typhoid IgG and IgM confirmation of PCR diagnostics

**Fig 4:**
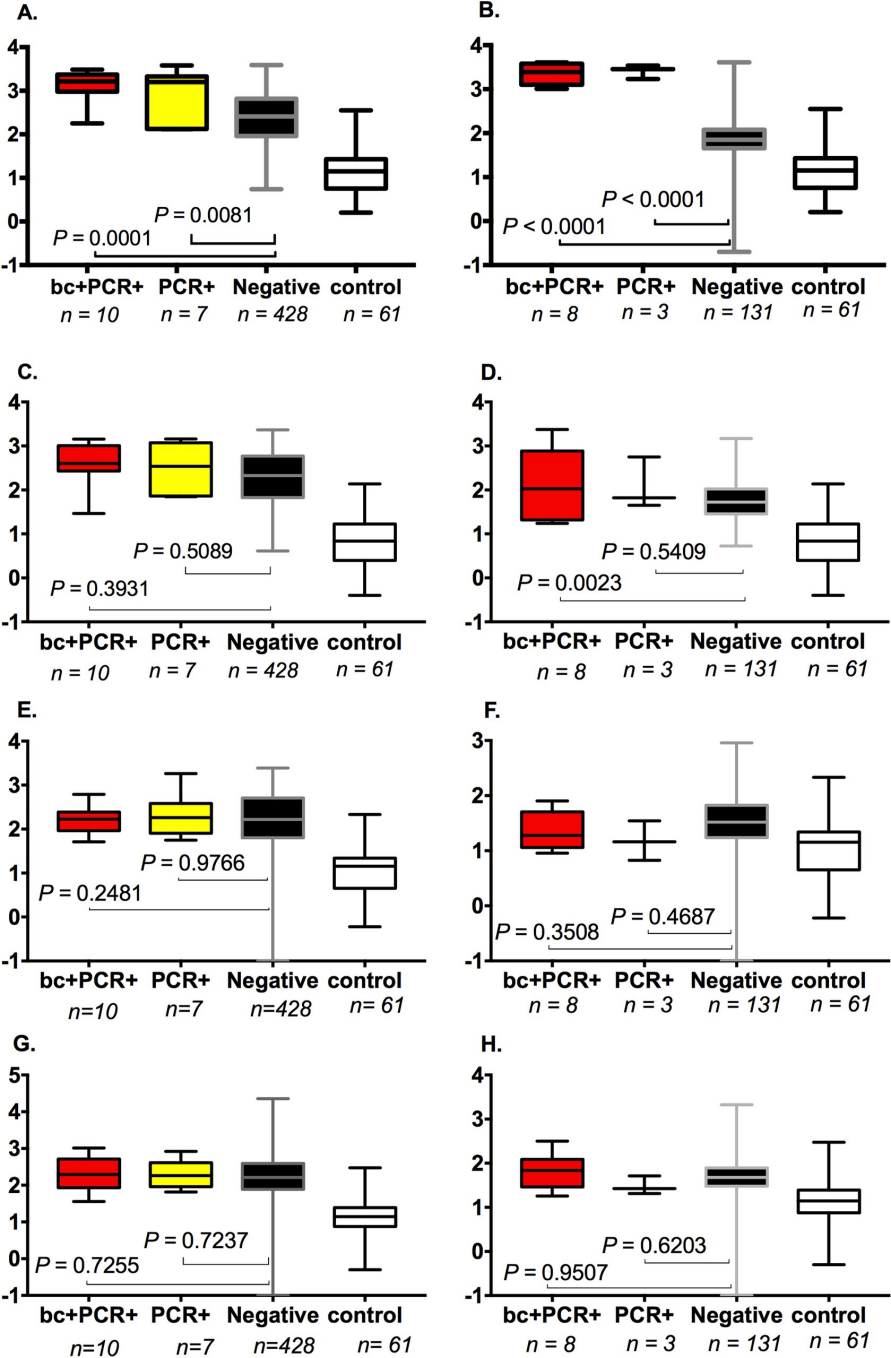
**Distribution of IgG antibody titres during acute infection (A, C, E, G) and in convalescence (B, D, F, H).** IgG antibody titres were measured against four antigens (STY1498 in A & B, STY1479 in C & D, STY1886 in E & F, & Vi in G & H) across distinct groups of children. The groups are **bc+PCR+** (cases that had S. Typhi infection confirmed by both blood culture and PCR (or blood culture alone), in acute infection [age 14 to 45, median 37 months], and in convalescence [age 14 to 45, median 33 months]), **PCR+** (S. Typhi infection confirmed by PCR only, in cute infection [age 8 to 46, median 22 months] and in convalescence [age 22 to 46, median 40 months]), **Negative** (febrile but negative for typhoid on blood culture and PCR, in acute infection [age 1 to 48, median 13.5 months] and in convalescence [age 1 to 45, median 16 months]), and **Control** (afebrile healthy controls [age 0 to 52.5, median 10.2 months]).

**Fig 5:**
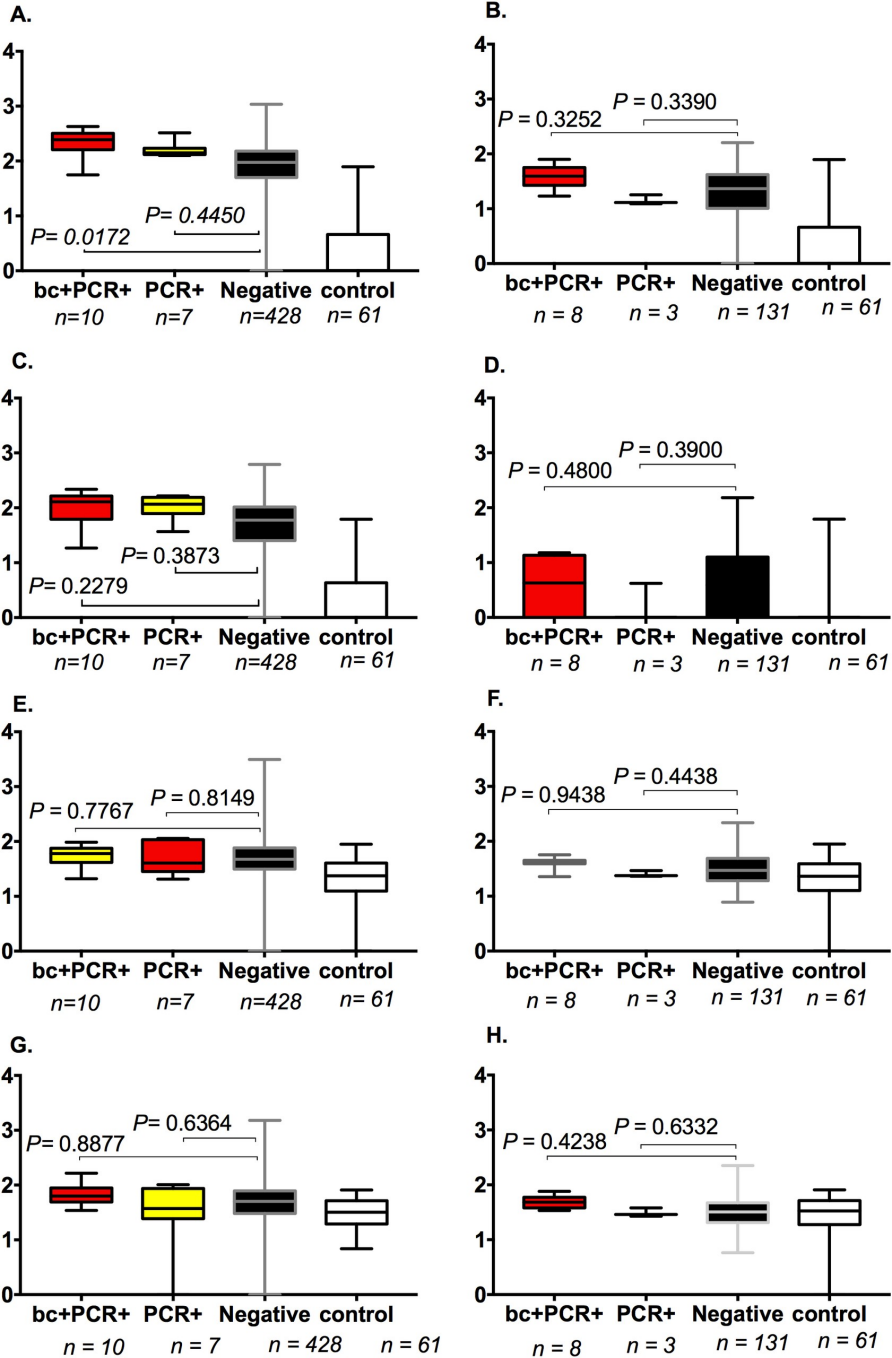
**Distribution of IgM antibody titres during acute infection (A, C, E, G) and in convalescence (B, D, F, H).** IgM antibodies were measured against four antigens (STY1498 in A & B, STY1479 in C & D, STY1886 in E & F & Vi in G &H) across distinct groups of children. The groups are **bc+PCR+** (cases that had S. Typhi infection confirmed by both blood culture and PCR (or blood culture alone), in acute infection [age 14 to 45, median 37 months], and in convalescence [age 14 to 45, median 33 months]), **PCR+** (S. Typhi infection confirmed by PCR only, in cute infection [age 8 to 46, median 22 months] and in convalescence [age 22 to 46, median 40 months]), **Negative** (febrile but negative for typhoid on blood culture and PCR, in acute infection [age 1 to 48, median 13.5 months] and in convalescence [age 1 to 45, median 16 months]), and **Control** (afebrile healthy controls [age 0 to 52.5, median 10.2 months]).

Serum was sampled from 445 children at the time of illness; 142 provided additional plasma sample six weeks after the admission date. All the 445 febrile children (regardless of presence or absence of *S*. Typhi infection) had significantly elevated IgM and IgG titres against all four antigens, compared to healthy controls (n = 61) (*P* range <0.0001 to 0.0394) (S3 Fig). IgM and IgG responses in acute typhoid infection, confirmed by both blood culture and PCR (or blood culture alone) (n = 10) against STY1498 were significantly elevated in comparison to responses in febrile children with all other non-typhoid illnesses (n = 428) (IgM, *P* = 0.0172; IgG, *P* = 0.0001) (Figs 4 and 5). The concentration of IgG against STY1498 in convalescent plasma from children who previously had a negative blood culture but were PCR amplification positive for *S*. Typhi were significantly higher than in children with non-typhoid illnesses (*P*< 0.0001) (Fig 4). There was no significant difference in IgM or IgG titres (*P*> = 0.05) against STY1479, STY1886 or Vi between children with typhoid infection and those with other illnesses (Figs 4 and 5). Further, neither multidimensional scaling nor principal component analysis, when run on both IgM and IgG titres for all four antigens, was able to clearly separate Salmonella positive from negative cases on blood culture and / or PCR (S4 Fig).

## Discussion

The precise epidemiology of invasive Salmonella infections remains elusive in many resource-limited settings [33, 34]. We have utilised a combination of blood culture and real-time PCR with pre-enrichment to improve ascertainment of *S*. Typhi and *S*. Typhimurium bacteraemia in young children by 62% and 94% respectively.

In sub-Saharan Africa, including Malawi, invasive nontyphoidal Salmonellae (iNTS) has been widely reported in children less than five years old [5, 6], leading to preventive strategies being targeted towards this age group [35]. In the context of multiple reports of declining incidence of iNTS in the region [1, 36], our findings suggest that the residual burden of this high mortality bloodstream infection may be greater than supposed. In contrast to iNTS, the conventional understanding has been that typhoid fever is a disease of older children and adults [25, 37, 38]. Our data suggests a considerable hidden burden in children 0–4 years who present with non-specific clinical features. We speculate that an even greater number of cases are presenting to community health centres and receiving only partially effective antibiotics.

The performance of both real-time PCR with culture pre-enrichment and blood culture is likely to have been limited by small volumes of blood available from young children and prior antibiotic use [39]. Nonetheless, real-time PCR with pre-enrichment did identify additional cases to blood culture with few false negatives. Reported prior use of antibiotics, which is expected to impact negatively both real-time PCR with culture pre-enrichment and blood culture, was found not to have affected detection of invasive salmonella infection. However, the unreliable nature of reported use of antibiotics [40, 41] may mean that our findings may still be an underestimate of invasive Salmonellosis in young children.

To validate case ascertainment by pre-enrichment PCR in the context of a negative blood culture, we have used antibody-based diagnostics [42, 43], which are not dependent on bacterial concentration in the blood but can vary between populations [44]. In this study IgM and IgG antibody responses to *S*. Typhi antigens Vi and CdtB (STY1886) in Malawian children were not serodiagnostic as previously reported in Vietnamese [30] and Bangladeshi [31] populations. Only IgG and IgM responses to STY1498 (haemolysin gene, *hlyE*) separated S. Typhi cases confirmed by blood culture and PCR with culture pre-enrichment, from those with illness due to other infections. The serodiagnostic capacity of STY1498 in Malawian children was evident in sera from active infection, as described for Vietnamese population [30], and also in convalescent plasma screening. STY1498 serology also validated the blood culture negative/ PCR negative results.

In conclusion, the combination of real-time PCR with culture pre-enrichment with blood culture improved case ascertainment among children aged between 0 and 4 years. These data highlight the hidden burden of invasive Salmonellosis in young children and support the targeting of pre-school children for typhoid vaccine prevention. The recent roll-out of a typhoid conjugate vaccine trial in Malawi and subsequent implementation will likely avert a greater burden of disease than previously reported. However, impact assessment of the vaccine may be affected by poor sensitivity of blood culture which is the primary endpoint for the vaccine trial. Future vaccine trial designs should consider using a combination of blood culture and real-time PCR with culture pre-enrichment as part of the evaluation of the full impact of the intervention.

## Supporting information

S1 STROBE A check list of items relevant to this report of a prospective diagnostic cohort study. (DOC)

S1 Fig **Receiver operating curve characteristic for the pan-primer and S. Typhi specific primer in (a) and for the pan-primer and S. Typhimurium specific primer in (b).** Generated from CT-values plotted against the blood culture ‘reference standard results’.
(TIF)

S2 Fig Distribution of CT-values according to volume of blood sample pre-enriched for PCR. Graphs show distribution according to (a) pan-primer invA, (b) S. Typhi specific primer staG, (c) S. Typhimurium specific primer fliC. Red triangle = PCR positive for S. Typhi, Blue rectangle = PCR positive for S. Typhimurium, and Black circle = PCR negative.
(TIF)

S3 Fig IgM and IgG antibody responses to four antigens (STY1886, STY1479, STY1498 and VI) in febrile children and healthy controls. The graph shows significantly higher IgM (blue) and IgG (red) levels in febrile children (n = 445) than IgM (clear) and IgG (purple) levels in healthy community controls (n = 61).
(TIF)

S4 Fig Multidimensional scaling and Principal component analysis of IgM and IgG responses to all four antigens (STY1886, STY1479, STY1498 and VI). (**1**) is IgM and IgG responses where blood culture and PCR were positive for Salmonella. (**3**) is IgG and IgM responses where blood culture was positive for Salmonella and PCR was negative. (**4**) is IgG and IgM responses where PCR was positive for Salmonella and blood culture was negative. (**7**) is IgG and IgM responses where both blood culture and PCR were negative for Salmonella. **Ctrl** are healthy controls.
(TIF)

S1 Table Concentrations of primers and probes for the PCR reaction. (PDF)

S2 Table Bacterial colony forming units per mL. Concentrations of bacteria serially diluted and DNA extracted to assess PCR non-specific amplification.
(PDF)

S3 Table Area under the curve for the receiver operating curve. The pan-primer and S. Typhi primer in **(a)** and the pan-primer and S. Typhimurium primer in **(b).**
(PDF)
