# Supplementary material for: Ascertaining the burden of invasive Salmonella disease in hospitalised febrile children aged under four years in Blantyre, Malawi
Source: PLoS Negl Trop Dis. 2019 Jul 17;13(7):e0007539. doi: 10.1371/journal.pntd.0007539 (PMC6663031; doi:10.1371/journal.pntd.0007539)
Supplement: S1 STROBE — (DOC) [file pntd.0007539.s001.doc]

STROBE Statement—Checklist of items that should be included in reports of ***cohort studies***

|  | Item No | Recommendation |
| --- | --- | --- |
| **Title and abstract** | 1 | (*a*) Indicate the study’s design with a commonly used term in the title or the abstract  “This is a prospective diagnostic cohort study. The study design is indicated in the abstract” |
|  |  | (*b*) Provide in the abstract an informative and balanced summary of what was done and what was found  “The abstract covers the following  **Background**: Typhoid fever is endemic across sub-Saharan Africa. However, estimates of the burden of typhoid are undermined by insufficient blood volumes and lack of sensitivity of blood culture.  **Objective**: Here, we aimed to address this limitation by exploiting pre-enrichment culture followed by PCR, alongside routine blood culture to improve typhoid case detection  **Design**: Prospective diagnostic cohort study  **Setting**: We carried out a prospective diagnostic cohort study and enrolled children (aged 0-4 years) with non-specific febrile disease admitted to a tertiary hospital in Blantyre, Malawi from August 2014 to July 2016.  **Participants**: children (aged 0-4 years) with non-specific febrile disease admitted to a tertiary hospital in Blantyre, Malawi from August 2014 to July 2016.  **Measurements:** Blood was collected for culture (BC) and real-time PCR after a pre-enrichment culture in tryptone soy broth and ox-bile. DNA was subjected to PCR for *invA* (Pan-*Salmonella*), *staG* (*S*. Typhi), and *fliC* (*S.* Typhimurium) genes. A positive PCR was defined as *invA* plus either *staG* or *fliC* (CT<29). IgM and IgG ELISA against four *S*. Typhi antigens was also performed.  **Results:** In total, 643 children (median age 1.3 years) with nonspecific febrile disease were enrolled; 31 (4.8%) were BC positive for *Salmonella* (n=13 *S*. Typhi, n=16 *S*. Typhimurium, and n=2 *S*. Enteritidis). Pre-enrichment culture of blood followed by PCR identified a further 8 *S*. Typhi and 15 *S*. Typhimurium positive children. IgM and IgG titres to the S. Typhi antigen STY1498 (haemolysin) were significantly higher in children that were PCR positive but blood culture negative compared to febrile children with all other non-typhoid illnesses. The addition of pre-enrichment culture and PCR increased the case ascertainment of invasive *Salmonella* disease in children by 62-94%.  **Conclusions:** These data support recent burden estimates that highlight the insensitivity of blood cultures and support the targeting of pre-school children for typhoid vaccine prevention in Africa. Blood culture with real-time PCR following pre-enrichment should be used to further refine estimates of vaccine effectiveness in typhoid vaccine trials.” |
| Introduction | | |
| Background/rationale | 2 | Explain the scientific background and rationale for the investigation being reported  **“The background/rationale is provided in the manuscript as follows:**  Both *Salmonella* Typhi and nontyphoidal Salmonellae remain prominent contributors to the large burden of bloodstream infection (BSI) in sub-Saharan Africa (sSA) [1-4]. Until recently nontyphoidal serovars *Salmonella* Typhimurium and *Salmonella* Enteritidis were the most prevalent in sSA, mainly affecting young children and HIV-infected adults [5-7]. Outbreaks of typhoid fever are now being reported across sSA [1, 8-11], largely caused by a multidrug resistant *S.* Typhi genotype 4.3.1 [12-14]. In Malawi where surveillance for bloodstream infections has been conducted for over 20 years, *S*. Typhi has become one of the commonest blood culture isolates amongst hospitalized febrile adults and children [1, 15]. In this context, ineffective, commonly available antimicrobials and inadequate diagnostic tools with poor sensitivity and results turn-around time, hamper the identification, management and control of both iNTS and typhoid fever.  Blood culture identifies 40 to 80% of cases of invasive Salmonella disease [16]. Limitations of culture arise due to low numbers of bacteria in blood, requiring large volume samples that are not feasible from young children [17]. Prior antimicrobial use may further compromise the diagnostic yield. Currently available, commercial serological tests detecting antibody against *S*. Typhi antigens have limited sensitivity and poor specificity [18-20]. A number of PCR methods for diagnosis of *Salmonella* have been developed. The inclusion of a culture step prior to nucleic acid amplification has been suggested to increase sensitivity [21, 22], but this has not been evaluated in the field.  Studies from countries in Asia with endemic typhoid suggest a considerable disease burden in young children, including children aged under 4 years [3, 23-25]. If this is also the case in sSA, this could influence the age at which vaccination might be implemented within the National vaccination expanded programme on immunisation [26]. In this study, we adopted real-time PCR with a pre-enrichment culture step alongside standard blood culture to improve case ascertainment of invasive *Salmonella* disease in children aged under 4 years in Malawi.” |
| Objectives | 3 | State specific objectives, including any prespecified hypotheses  “In this study, we adopted real-time PCR with a pre-enrichment culture step alongside standard blood culture to improve case ascertainment of invasive *Salmonella* disease in children aged under 4 years in Malawi.” |
| Methods | | |
| Study design | 4 | Present key elements of study design early in the paper  **“Recruitment and follow-up are described as follows:**  Recruitment of participants into the study was nested within the pre-existing sentinel surveillance for bacterial infections at Queen Elizabeth Central Hospital (QECH) and the Malawi-Liverpool-Wellcome Trust, Blantyre, Malawi. Children with non-specific febrile illness (temperature ≥38°C or a history of fever) aged between 0 and 4 years old presenting to QECH were recruited. QECH is the largest government hospital in Malawi, providing free healthcare to the district of Blantyre with a population of approximately 1.3 million and referrals from the Southern region [27]. Recruitment was from August 2014 to July 2016 covering three dry seasons and two rainy seasons. 5 mL blood was drawn from each child; 2 mL for culture, 2 mL for real-time PCR and 1 mL for serology. At six weeks in convalescence further 1 mL of blood was drawn and plasma was extracted for serology. Unless invasive bacterial infection was strongly suspected, children with a positive RDT or malaria blood film were not enrolled into the study.” |
| Setting | 5 | Describe the setting, locations, and relevant dates, including periods of recruitment, exposure, follow-up, and data collection  “QECH is the largest government hospital in Malawi, providing free healthcare to the district of Blantyre with a population of approximately 1.3 million and referrals from the Southern region [27]. Recruitment was from August 2014 to July 2016 covering three dry seasons and two rainy seasons. 5 mL blood was drawn from each child; 2 mL for culture, 2 mL for real-time PCR and 1 mL for serology. At six weeks in convalescence further 1 mL of blood was drawn and plasma was extracted for serology. Unless invasive bacterial infection was strongly suspected, children with a positive RDT or malaria blood film were not enrolled into the study.” |
| Participants | 6 | (*a*) Give the eligibility criteria, and the sources and methods of selection of participants. Describe methods of follow-up  “Recruitment of participants into the study was nested within the pre-existing sentinel surveillance for bacterial infections at Queen Elizabeth Central Hospital (QECH) and the Malawi-Liverpool-Wellcome Trust, Blantyre, Malawi. Children with non-specific febrile illness (temperature ≥38°C or a history of fever) aged between 0 and 4 years old presenting to QECH were recruited……”  “……Unless invasive bacterial infection was strongly suspected, children with a positive RDT or malaria blood film were not enrolled into the study.” |
|  |  | (*b*) For matched studies, give matching criteria and number of exposed and unexposed  N/A |
| Variables | 7 | Clearly define all outcomes, exposures, predictors, potential confounders, and effect modifiers. Give diagnostic criteria, if applicable  **“Blood culture processing**  Blood cultures were processed using the BacT/Alert automated system (Biomerieux, France), at the Malawi-Liverpool-Wellcome Trust Clinical Research Laboratories [1]. Bacteria were isolated and identified using previously described standard microbiological procedures [1]. A minimum of four days, including sub-culturing, biochemical testing and latex agglutination testing, was necessary to generate a definitive report of the diagnosis.”  “Establishing a cycle threshold cut-off  A positive PCR result was assigned to a sample only if the pan-*Salmonella* (*invA*) amplification was simultaneously positive with either one of the serovar-specific amplifications (*fliC* or *staG*) within a specified cycle threshold (CT) cut-off. The CT-value cut-off was determined using a combination of three approaches. Firstly, the primer pairs were tested against DNA extracted from serially diluted cultures of *S.* Typhi, *S.* Typhimurium, *Staphylococcus aureus*, *E. coli*, *Klebsiella pneumoniae*, *Micrococcus* spp., *Bacillus*. Spp, and coagulase negative *Staphylococcus*. Five replicates were prepared for each organism. A cut-off was set to exclude all CT-values observed for non-specific target amplifications. Secondly, the limit of target detection was established for the three primer pairs by running the real-time PCR assay on five replicates of serially diluted cultures of *S*. Typhi and *S*. Typhimurium. Thirdly, a receiver operating characteristic (ROC) curve was constructed to assess the cut-off value that maximises sensitivity and specificity of the PCR amplification, considering the blood culture result as a true positive.”  ***“S.* Typhi serology**  Serology for *S*. Typhi (Table 2), with previously identified serodiagnostic antigens [29, 30] and S. Typhi Vi polysaccharide antigen [31], was conducted to validate the PCR amplification data in blood culture negative samples. Sera from febrile children acutely ill and plasma at six weeks in convalescence were analysed. Archived serum samples from healthy Malawian children aged 0 to 4 years (median age 10 months) were included in the analysis as controls [32]. IgM and IgG titres in acute and convalescent plasma samples and in healthy control sera were measured using a previously described ELISA with 3 purified protein antigens STY1498 (Haemolysin), STY1479 (a possible ATP binding protein), STY1886 (cytolethal distending toxin subunit B homolog) and S. Typhi Vi polysaccharide antigen.” |
| Data sources/ measurement | 8* | Clearly define all outcomes, exposures, predictors, potential confounders, and effect modifiers. Give diagnostic criteria, if applicable  **“Blood culture processing**  Blood cultures were processed using the BacT/Alert automated system (Biomerieux, France), at the Malawi-Liverpool-Wellcome Trust Clinical Research Laboratories [1]. Bacteria were isolated and identified using previously described standard microbiological procedures [1]. A minimum of four days, including sub-culturing, biochemical testing and latex agglutination testing, was necessary to generate a definitive report of the diagnosis.”  **“Pre-enrichment**  A modified pre-enrichment procedure for *Salmonella* was adopted [28]. A maximum of 2 ml of blood was added to 8 ml of tryptone soy broth mixed with 3% Ox-gall powder (TSB/Ox-gall) and incubated overnight at 37°C. Blood samples with <2 ml were added to 8 mL TSB/Ox-gall medium, topped up to 10 mL with sterile distilled water.”  **“Establishing a cycle threshold cut-off**  A positive PCR result was assigned to a sample only if the pan-*Salmonella* (*invA*) amplification was simultaneously positive with either one of the serovar-specific amplifications (*fliC* or *staG*) within a specified cycle threshold (CT) cut-off. The CT-value cut-off was determined using a combination of three approaches. Firstly, the primer pairs were tested against DNA extracted from serially diluted cultures of *S.* Typhi, *S.* Typhimurium, *Staphylococcus aureus*, *E. coli*, *Klebsiella pneumoniae*, *Micrococcus* spp., *Bacillus*. Spp, and coagulase negative *Staphylococcus*. Five replicates were prepared for each organism. A cut-off was set to exclude all CT-values observed for non-specific target amplifications. Secondly, the limit of target detection was established for the three primer pairs by running the real-time PCR assay on five replicates of serially diluted cultures of *S*. Typhi and *S*. Typhimurium. Thirdly, a receiver operating characteristic (ROC) curve was constructed to assess the cut-off value that maximises sensitivity and specificity of the PCR amplification, considering the blood culture result as a true positive.”  ***“S.* Typhi serology**  Serology for *S*. Typhi (Table 2), with previously identified serodiagnostic antigens [29, 30] and S. Typhi Vi polysaccharide antigen [31], was conducted to validate the PCR amplification data in blood culture negative samples. Sera from febrile children acutely ill and plasma at six weeks in convalescence were analysed. Archived serum samples from healthy Malawian children aged 0 to 4 years (median age 10 months) were included in the analysis as controls [32]. IgM and IgG titres in acute and convalescent plasma samples and in healthy control sera were measured using a previously described ELISA with 3 purified protein antigens STY1498 (Haemolysin), STY1479 (a possible ATP binding protein), STY1886 (cytolethal distending toxin subunit B homolog) and S. Typhi Vi polysaccharide antigen.” |
| Bias | 9 | Describe any efforts to address potential sources of bias  “The CT-value cut-off was determined using a combination of three approaches. Firstly, the primer pairs were tested against DNA extracted from serially diluted cultures of *S.* Typhi, *S.* Typhimurium, *Staphylococcus aureus*, *E. coli*, *Klebsiella pneumoniae*, *Micrococcus* spp., *Bacillus* spp., and coagulase negative *Staphylococcus*. Five replicates were prepared for each organism. A cut-off was set to exclude all CT-values observed for non-specific target amplifications. Secondly, the limit of target detection was established for the three primer pairs by running the real-time PCR assay on five replicates of serially diluted cultures of *S*. Typhi and *S*. Typhimurium. Thirdly, a receiver operating characteristic (ROC) curve was constructed to assess the cut-off value that maximises sensitivity and specificity of the PCR amplification, considering the blood culture result as a true positive.” |
| Study size | 10 | Explain how the study size was arrived at  In the study protocol sample size is determined as follows:  **Sample Size**: Sample size is based on precision of estimation of the prevalence assuming an asymptotically large sampling population, and using the following formula for the precision:  ME = z * sqrt{p*(1-p)/(n-1)}  Where ME is the margin of error; z is the critical value from the Z-distribution (for 95% confidence this is 1.96); p is the prevalence; and n is the sample size.The greater the precision, the greater the test sensitivity and specificity. Greater sample sizes are required for more precise estimation.    **Table 1**: Sample size by prevalence and precision   |  | **Margin of Error *** | | | | | --- | --- | --- | --- | --- | | **True prevalence (%)** | **15%** | **10%** | **5%** | **2%** | | **5** |  |  | 73 | 457 | | **10** |  | 36 | 139 | 865 | | **20** | 28 | 62 | 246 | 1537 | | **30** | 36 | 81 | 323 | 2017 | | **40** | 41 | 93 | 369 | 2305 | | **50** | 43 | 97 | 385 | 2401 | | **60** | 41 | 93 | 369 | 2305 |   * The ME is half the span of the confidence boundary, e.g. if true prevalence is 20% and margin of error is 5%, then the test will detect 20% prevalence with 95% confidence interval spanning (15% to 25%)  The number of children <2 years with RDT negative non-specific febrile illness presenting to paediatric Accident & Emergency at QECH is about 1000 per year. Thus with recruitment over 12 months we will have power to detect a broad range of true prevalence with high precision. |
| Quantitative variables | 11 | Explain how quantitative variables were handled in the analyses. If applicable, describe which groupings were chosen and why  **“Establishing a cycle threshold cut-off**  A positive PCR result was assigned to a sample only if the pan-*Salmonella* (*invA*) amplification was simultaneously positive with either one of the serovar-specific amplifications (*fliC* or *staG*) within a specified cycle threshold (CT) cut-off. The CT-value cut-off was determined using a combination of three approaches. Firstly, the primer pairs were tested against DNA extracted from serially diluted cultures of *S.* Typhi, *S.* Typhimurium, *Staphylococcus aureus*, *E. coli*, *Klebsiella pneumoniae*, *Micrococcus* spp., *Bacillus* spp., and coagulase negative *Staphylococcus*. Five replicates were prepared for each organism. A cut-off was set to exclude all CT-values observed for non-specific target amplifications. Secondly, the limit of target detection was established for the three primer pairs by running the real-time PCR assay on five replicates of serially diluted cultures of *S*. Typhi and *S*. Typhimurium. Thirdly, a receiver operating characteristic (ROC) curve was constructed to assess the cut-off value that maximises sensitivity and specificity of the PCR amplification, considering the blood culture result as a true positive.” |
| Statistical methods | 12 | (a) Describe all statistical methods, including those used to control for confounding  **“Statistical analysis**  Fisher’s exact test and Chi-squared test were used to determine whether clinical characteristics (fever, vomiting and diarrhoea), hospital admission and reported prior use of antibiotics were related to detection of Salmonella by both culture and PCR. Statistical significance was defined as p < 0.05, although exact p-values were reported. Pearson’s correlation coefficient was used to analyse the association between blood sample volume and cycle threshold value on PCR. Antibody (IgG and IgM) titre differences between *S*. Typhi positive and negative groups were log-transformed and tested using two-tailed two-sample t-tests and we also looked for differential clustering between these 2 groups using multidimensional scaling and principal component analysis. Analyses were performed using IBM Corp. Released 2011. IBM SPSS Statistics for Windows, Version 20.0. Armonk, NY: IBM Corp, GraphPad prism version 7.0 and R v3.5.1. |
|  |  | (*b*) Describe any methods used to examine subgroups and interactions  N/A |
|  |  | (*c*) Explain how missing data were addressed  Missing data were excluded from analysis |
|  |  | (*d*) If applicable, explain how loss to follow-up was addressed |
|  |  | (*e*) Describe any sensitivity analyses |
| Results | | |
| Participants | 13* | (a) Report numbers of individuals at each stage of study—eg numbers potentially eligible, examined for eligibility, confirmed eligible, included in the study, completing follow-up, and analysed  Samples were tested for IgG and IgM against FOUR S.Typhi antigens  **142** with either Salmonella growth or any PCR CT-value provided a plasma sample at six weeks in convalescence  **19** children were excluded; 6 had no blood culture result; 4 had no PCR sample; and 9 had inconclusive PCR results on re-testing.  Children screened  N = **1105**  Febrile children recruited into the study  n = **662**  Febrile children with blood culture and PCR Samples collected and processed  n = **643**  **445** febrile children provided serum for typhoid serology |
|  |  | (b) Give reasons for non-participation at each stage |
|  |  | (c) Consider use of a flow diagram |
| Descriptive data | 14* | (a) Give characteristics of study participants (eg demographic, clinical, social) and information on exposures and potential confounders  “Study population  A total of 661 children aged less than 4 years (median age 15.9 months; range 1 – 48 months; 357 males), with non-specific febrile disease were recruited at QECH over the study period. Eighteen children were excluded from the analysis; 5 had no blood culture sample taken, 4 had no PCR sample, and 9 had inconclusive PCR results on re-testing.  The baseline characteristics of the 643 children in these analyses are presented in Table 3.”   | **Characteristic** | **Children ≤ 4yrs**  **n (%)** | | --- | --- | | Number | 643 | | Sex, male | 351 (54.6) | | Age, months, median [IQR] | 15.8 [8.9 – 28] | | 1 - 8.9 | 165 (25.5) | | 9 - 16.9 | 172 (26.7) | | 17 - 24.9 | 114 (17.7) | | 25 - 48 | 192 (29.7) | | Vomiting | 261/641 (40.7) | | Diarrhoea | 227/641 (35.4) | | Malaria positive by RDT or Blood Film Microscopy | 20/640 (3.1) | | Recruitment by season, wet | 360 (56.0) | | Hospital admission | 252/639 (39.4) | | Prior reported antibiotic use | 288/642 (44.9) | |
|  |  | (b) Indicate number of participants with missing data for each variable of interest  “Study population  A total of 661 children aged less than 4 years (median age 15.9 months; range 1 – 48 months; 357 males), with non-specific febrile disease were recruited at QECH over the study period. Eighteen children were excluded from the analysis; 5 had no blood culture sample taken, 4 had no PCR sample, and 9 had inconclusive PCR results on re-testing.” |
|  |  | (c) Summarise follow-up time (eg, average and total amount) |
| Outcome data | 15* | Report numbers of outcome events or summary measures over time  Table 5. PCR performance in relation to Blood culture  **“Clinical characteristics, antimicrobial use and *Salmonella* detection**  Non-specific clinical presentations including fever, vomiting and diarrhoea were common and there was no association between clinical signs or symptoms and detection of either *S.* Typhi or *S*. Typhimurium infection (*P* range 0.27 to 0.79). However, hospital admission was more frequent in children with *S*. Typhimurium infection (23/31; 74.1%) than those with typhoid fever (8/21; 38.0%) (*p* = 0.02). Reported prior-antibiotic use (288/642; 44.9%) was not significantly associated with lack of growth of either *S*. Typhi (*P* = 0.22) or nontyphoid Salmonella (*P =* 0.93).”  **“Real-time PCR and sample volume**  Blood sample volumes were recorded in 619 of the 643 recruited children. The targeted volume of blood (2 mL) for real-time PCR was achieved in 100 /619 (16.2%) children. The minimum volume of blood sample collected for PCR after venesection for blood culture was 100µl (median = 1,200µl, IQR 1,000µl – 1,725µl). There was no correlation between the CT value and the volume of the pre-enriched blood sample for the pan-primer (*P* = 0.35, *r* = .05), *S*. Typhi specific primer (*P* = 0.59, *r* = .04), and *S*. Typhimurium specific primer (*P* = 0.13, *r* = .10) (S2 Fig).”  “**Typhoid IgG and IgM confirmation of PCR diagnostics**  Serum was sampled from 445 children at the time of illness; 142 provided additional plasma sample six weeks after the admission date. All the 445 febrile children (regardless of presence or absence of *S*. Typhi infection) had significantly elevated IgM and IgG titres against all four antigens, compared to healthy controls (n= 61) (*P* range <0.0001 to 0.0394) (S3 Fig). IgM and IgG responses in acute typhoid infection, confirmed by both blood culture and PCR (or blood culture alone) (n= 10) against STY1498 were significantly elevated in comparison to responses in febrile children with all other non-typhoid illnesses (n= 428) (IgM, *p*= 0.0172; IgG, *P*= 0.0001) (Figures 3 and 4). The concentration of IgG against STY1498 in convalescent plasma from children who previously had a negative blood culture but were PCR amplification positive for *S*. Typhi were significantly higher than in children with non-typhoid illnesses (*P*< 0.0001) (Fig 3). There was no significant difference in IgM or IgG titres (*P*>=0.05) against STY1479, STY1886 or Vi between children with typhoid infection and those with other illnesses (Figs 3 and 4). Further, neither multidimensional scaling nor principal component analysis, when run on both IgM and IgG titres for all four antigens, was able to clearly separate Salmonella positive from negative cases on blood culture and / or PCR (S4 Fig).” |
| Main results | 16 | (*a*) Give unadjusted estimates and, if applicable, confounder-adjusted estimates and their precision (eg, 95% confidence interval). Make clear which confounders were  adjusted for and why they were included  N/A |
|  |  | (*b*) Report category boundaries when continuous variables were categorized  N/A |
|  |  | (*c*) If relevant, consider translating estimates of relative risk into absolute risk for a meaningful time period  N/A |
| Other analyses | 17 | Report other analyses done—eg analyses of subgroups and interactions, and sensitivity analyses  “……Further, neither multidimensional scaling nor principal component analysis, when run on both IgM and IgG titres for all four antigens, was able to clearly separate Salmonella positive from negative cases on blood culture and / or PCR (S4 Fig).” |
| Discussion | | |
| Key results | 18 | Summarise key results with reference to study objectives  “The precise epidemiology of invasive Salmonella infections remains elusive in many resource-limited settings [33, 34]. We have utilised a combination of blood culture and real-time PCR with pre-enrichment to improve ascertainment of *S*. Typhi and *S*. Typhimurium bacteraemia in young children by 62% and 94% respectively.  In sub-Saharan Africa, including Malawi, invasive nontyphoidal Salmonellae (iNTS) has been widely reported in children less than five years old [5, 6], leading to preventive strategies being targeted towards this age group [35]. In the context of multiple reports of declining incidence of iNTS in the region [1, 36], our findings suggest that the residual burden of this high mortality bloodstream infection may be greater than supposed. In contrast to iNTS, the conventional understanding has been that typhoid fever is a disease of older children and adults [25, 37, 38]. Our data suggests a considerable hidden burden in children 0-4 years who present with non-specific clinical features. We speculate that an even greater number of cases are presenting to community health centres and receiving only partially effective antibiotics.”  “To validate case ascertainment by pre-enrichment PCR in the context of a negative blood culture, we have used antibody-based diagnostics [42, 43], which are not dependent on bacterial concentration in the blood but can vary between populations [44]. In this study IgM and IgG antibody responses to *S*. Typhi antigens Vi and CdtB (STY1886) in Malawian children were not serodiagnostic as previously reported in Vietnamese [30] and Bangladeshi [31] populations. Only IgG and IgM responses to STY1498 (haemolysin gene, *hlyE*) separated S. Typhi cases confirmed by blood culture and PCR with culture pre-enrichment, from those with illness due to other infections. The serodiagnostic capacity of STY1498 in Malawian children was evident in sera from active infection, as described for Vietnamese population [30], and also in convalescent plasma screening. STY1498 serology also validated the blood culture negative/ PCR negative results.” |
| Limitations | 19 | Discuss limitations of the study, taking into account sources of potential bias or imprecision. Discuss both direction and magnitude of any potential bias  The performance of both real-time PCR with culture pre-enrichment and blood culture is likely to have been limited by small volumes of blood available from young children and prior antibiotic use [39]. Nonetheless, real-time PCR with pre-enrichment did identify additional cases to blood culture with few false negatives. Reported prior use of antibiotics, which is expected to impact negatively both real-time PCR with culture pre-enrichment and blood culture, was found not to have affected detection of invasive salmonella infection. However, the unreliable nature of reported use of antibiotics [40, 41] may mean that our findings may still be an underestimate of invasive Salmonellosis in young children. |
| Interpretation | 20 | Give a cautious overall interpretation of results considering objectives, limitations, multiplicity of analyses, results from similar studies, and other relevant evidence  “In conclusion, the combination of real-time PCR with culture pre-enrichment with blood culture improved case ascertainment among children aged between 0 and 4 years. These data highlight the hidden burden of invasive Salmonellosis in young children and support the targeting of pre-school children for typhoid vaccine prevention. The recent roll-out of a typhoid conjugate vaccine trial in Malawi and subsequent implementation will likely avert a greater burden of disease than previously reported (REF). However, impact assessment of the vaccine may be affected by poor sensitivity of blood culture which is the primary endpoint (ref) for the vaccine trial. Future vaccine trial designs should consider using a combination of blood culture and real-time PCR with culture pre-enrichment as part of the evaluation of the full impact of the intervention” |
| Generalisability | 21 | Discuss the generalisability (external validity) of the study results  “These data highlight the hidden burden of invasive Salmonellosis in young children and support the targeting of pre-school children for typhoid vaccine prevention. The recent roll-out of a typhoid conjugate vaccine trial in Malawi and subsequent implementation will likely avert a greater burden of disease than previously reported (REF). However, impact assessment of the vaccine may be affected by poor sensitivity of blood culture which is the primary endpoint (ref) for the vaccine trial. Future vaccine trial designs should consider using a combination of blood culture and real-time PCR with culture pre-enrichment as part of the evaluation of the full impact of the intervention” |
| Other information | | |
| Funding | 22 | Give the source of funding and the role of the funders for the present study and, if applicable, for the original study on which the present article is based  “This study was funded through a Strategic Award for the MLW Clinical Research Programme from the Wellcome Trust UK.” |

*Give information separately for exposed and unexposed groups.

**Note:** An Explanation and Elaboration article discusses each checklist item and gives methodological background and published examples of transparent reporting. The STROBE checklist is best used in conjunction with this article (freely available on the Web sites of PLoS Medicine at http://www.plosmedicine.org/, Annals of Internal Medicine at http://www.annals.org/, and Epidemiology at http://www.epidem.com/). Information on the STROBE Initiative is available at http://www.strobe-statement.org.
