# Supplementary figures and images for: Ascertaining the burden of invasive Salmonella disease in hospitalised febrile children aged under four years in Blantyre, Malawi
Source: PLoS Negl Trop Dis. 2019 Jul 17;13(7):e0007539. doi: 10.1371/journal.pntd.0007539 (PMC6663031; doi:10.1371/journal.pntd.0007539)

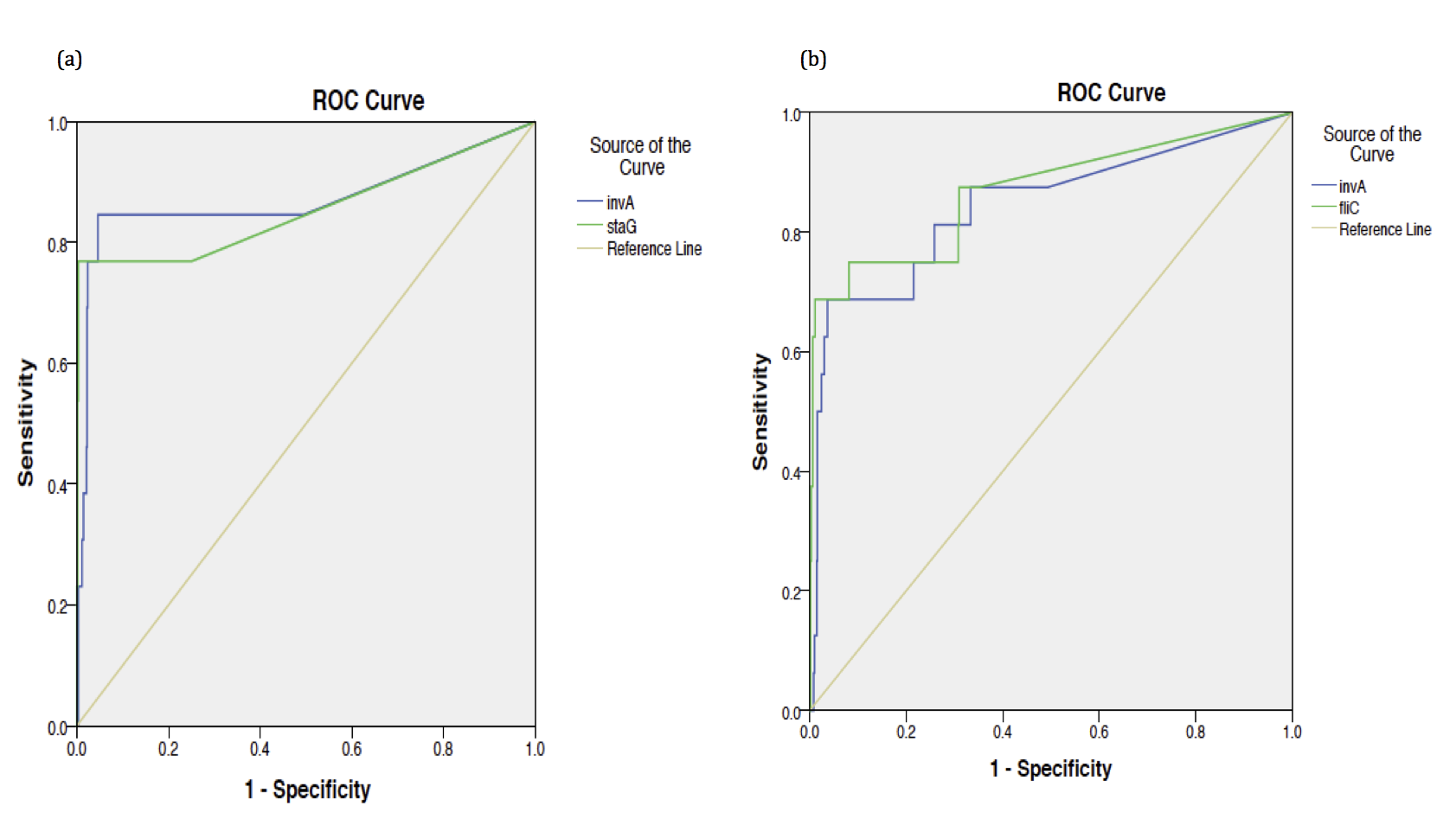

Supplement: S1 Fig — Receiver operating curve characteristic for the pan-primer and S. Typhi specific primer in (a) and for the pan-primer and S. Typhimurium specific primer in (b). Generated from CT-values plotted against the blood culture ‘reference standard results’. (TIF) [file pntd.0007539.s002.tif]

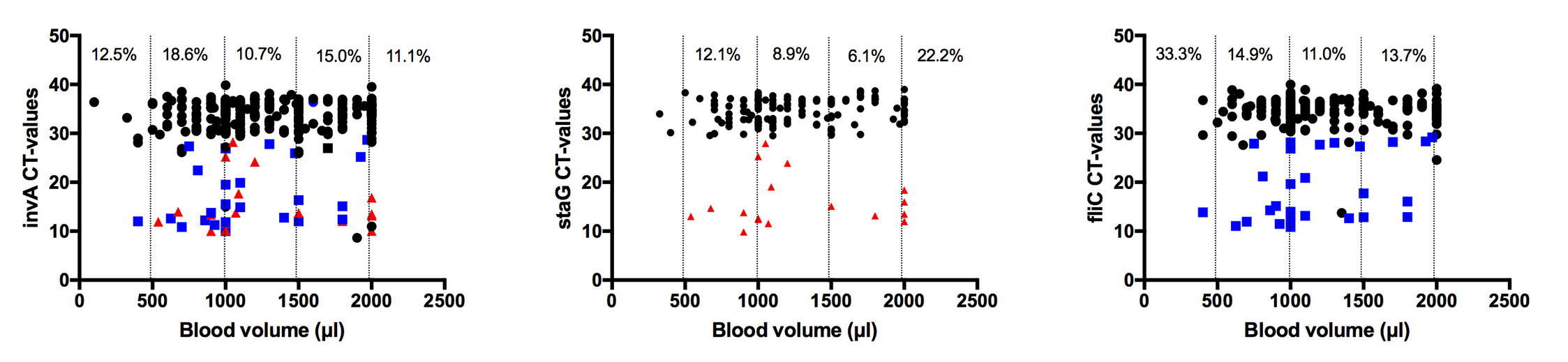

Supplement: S2 Fig — Graphs show distribution according to (a) pan-primer invA, (b) S. Typhi specific primer staG, (c) S. Typhimurium specific primer fliC. Red triangle = PCR positive for S. Typhi, Blue rectangle = PCR positive for S. Typhimurium, and Black circle = PCR negative. (TIF) [file pntd.0007539.s003.tif]

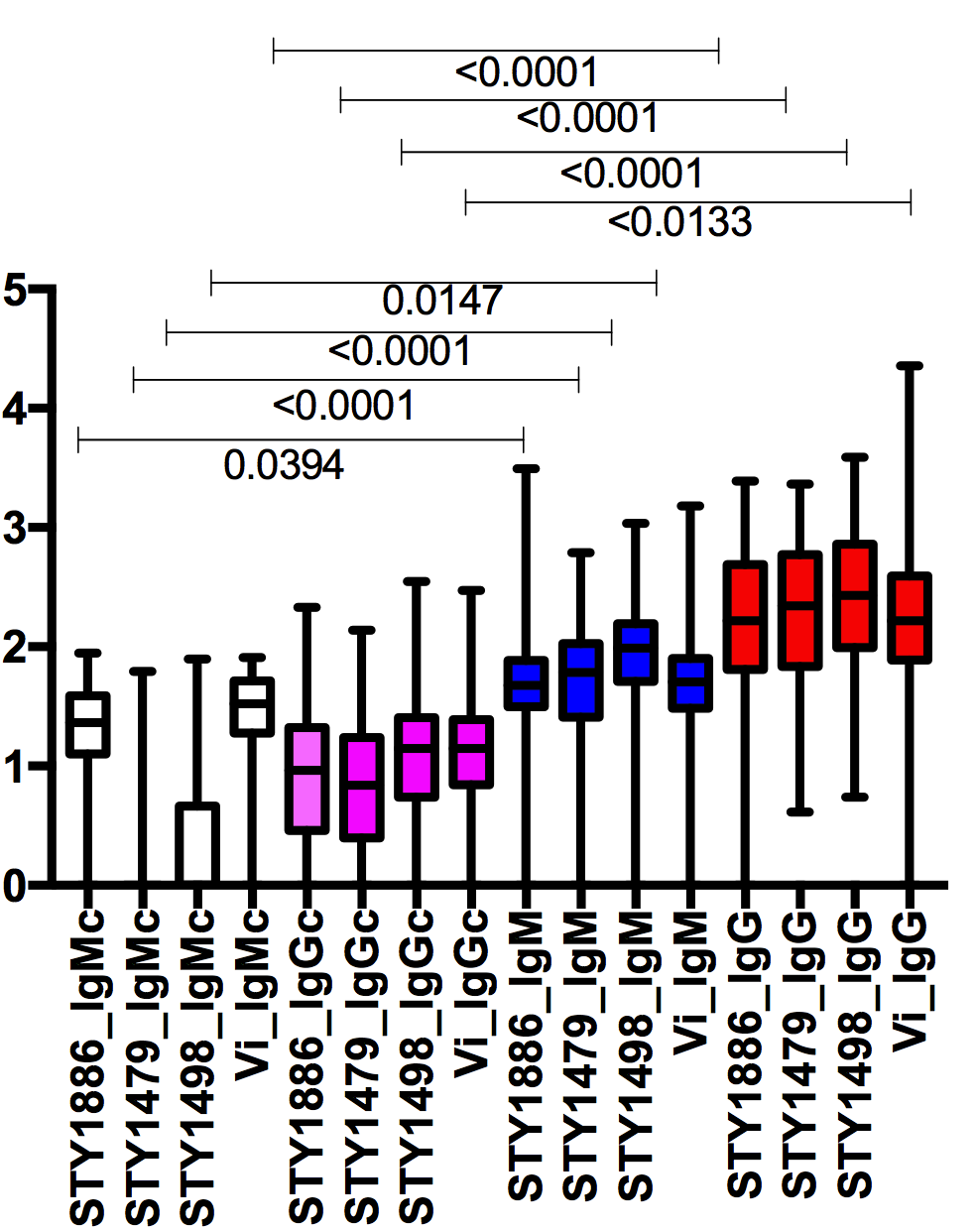

Supplement: S3 Fig — The graph shows significantly higher IgM (blue) and IgG (red) levels in febrile children (n = 445) than IgM (clear) and IgG (purple) levels in healthy community controls (n = 61). (TIF) [file pntd.0007539.s004.tif]

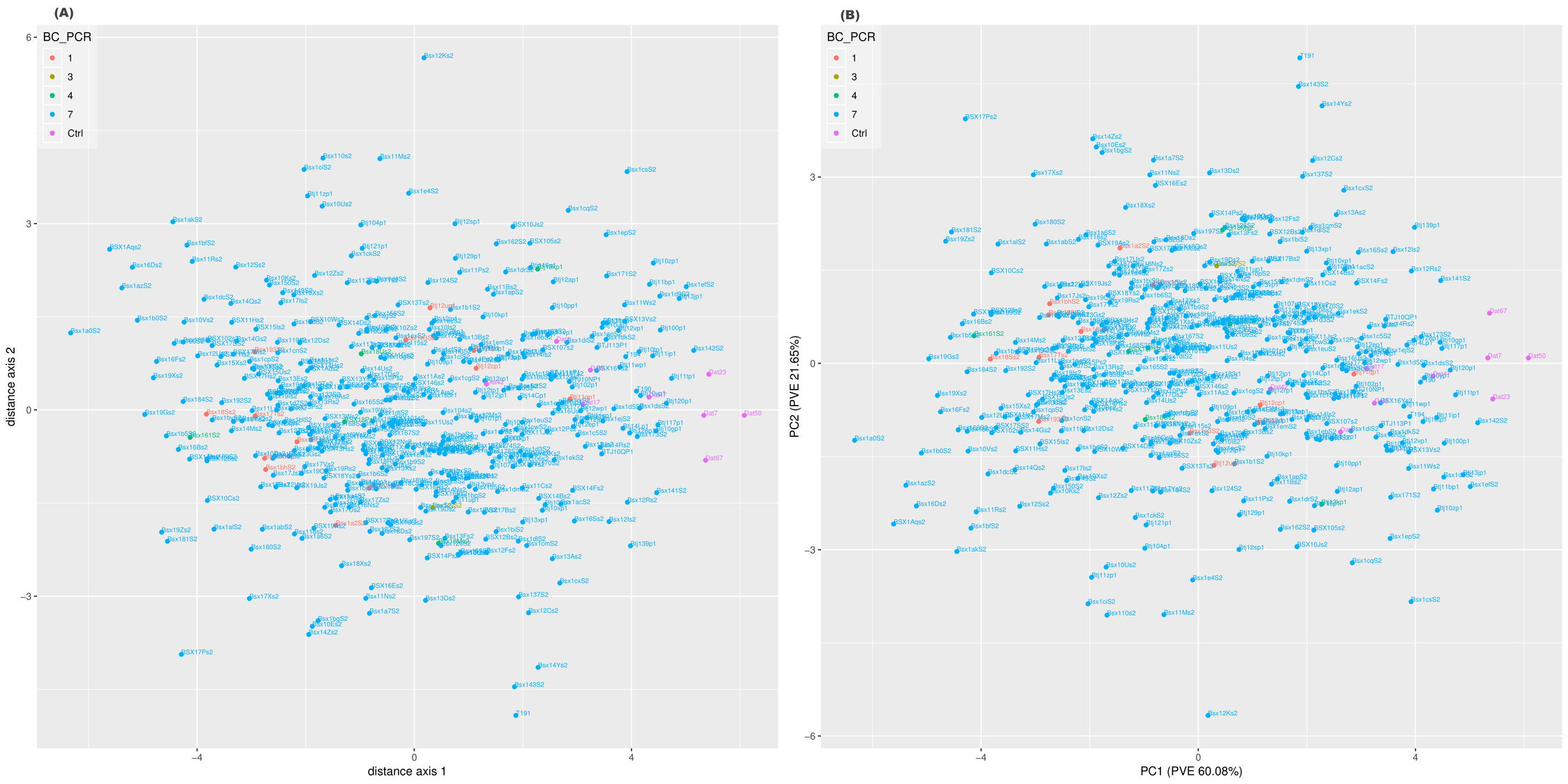

Supplement: S4 Fig — (1) is IgM and IgG responses where blood culture and PCR were positive for Salmonella. (3) is IgG and IgM responses where blood culture was positive for Salmonella and PCR was negative. (4) is IgG and IgM responses where PCR was positive for Salmonella and blood culture was negative. (7) is IgG and IgM responses where both blood culture and PCR were negative for Salmonella. Ctrl are healthy controls. (TIF) [file pntd.0007539.s005.tif]
