# Supplementary material for: Ascertaining the burden of invasive Salmonella disease in hospitalised febrile children aged under four years in Blantyre, Malawi
Source: PLoS Negl Trop Dis. 2019 Jul 17;13(7):e0007539. doi: 10.1371/journal.pntd.0007539 (PMC6663031; doi:10.1371/journal.pntd.0007539)
Supplement: S1 Table — (PDF) [file pntd.0007539.s006.pdf]

Supplementary data

2X reaction mix	12.5µl
Forward primer (50µM)	0.1µl
Reverse primer (50µM)	0.1µl
Probe (50µM)	0.1µl
Rox reference dye (25µM)	0.05µl
Nuclease-free water	7.15µl
**Total**	20µl
