# Supplementary material for: Ascertaining the burden of invasive Salmonella disease in hospitalised febrile children aged under four years in Blantyre, Malawi
Source: PLoS Negl Trop Dis. 2019 Jul 17;13(7):e0007539. doi: 10.1371/journal.pntd.0007539 (PMC6663031; doi:10.1371/journal.pntd.0007539)
Supplement: S2 Table — Concentrations of bacteria serially diluted and DNA extracted to assess PCR non-specific amplification. (PDF) [file pntd.0007539.s007.pdf]

Supplementary data

**Overnight cultures**	**Colony forming units**
S. Typhi	2.4*10^7
S. Typhimurium	1.4*10^8
Bacillus	2.0*10^7
Staphylococcus aureus	2.6*10^8
Klebsiella spp.	2.4*10^7
E.coli	1.0*10^8
Micrococci	2.7*10^7
