# Supplementary material for: Ascertaining the burden of invasive Salmonella disease in hospitalised febrile children aged under four years in Blantyre, Malawi
Source: PLoS Negl Trop Dis. 2019 Jul 17;13(7):e0007539. doi: 10.1371/journal.pntd.0007539 (PMC6663031; doi:10.1371/journal.pntd.0007539)
Supplement: S3 Table — The pan-primer and S. Typhi primer in (a) and the pan-primer and S. Typhimurium primer in (b). (PDF) [file pntd.0007539.s008.pdf]

Supplementary Data

(a)

Area Under the Curve

Test Result Variable(s)	Area	Std. Error[a]	Asymptotic Sig.[b]	Asymptotic 95% Confidence Interval	
				Lower Bound	Upper Bound
invA	.870	.075	.000	.723	1.000
fliD	.854	.078	.000	.701	1.000

(b)

Area Under the Curve

Test Result Variable(s)	Area	Std. Error[a]	Asymptotic Sig.[b]	Asymptotic 95% Confidence Interval	
				Lower Bound	Upper Bound
invA	.844	.063	.000	.721	.966
fliC	.869	.060	.000	.752	.986
